# Sijunzi, Lizhong, and Fuzilizhong Decoction Alleviate Nonalcoholic Fatty Liver Disease through Activation of PPAR Pathway

**DOI:** 10.1155/2020/6363748

**Published:** 2020-10-29

**Authors:** Jiayao Yang, Dongqing Tao, Wei Ma, Song Liu, Yan Liao, Lei Shu, Shu Zhang, Chenyu Li, Nianlong Du, Zhaohong Shi

**Affiliations:** ^1^Department of Gastroenterology, Wuhan Integrated TCM and Western Medicine Hospital, Wuhan, China; ^2^Department of Endocrinology, The Third People's Hospital of Hubei Province, Wuhan, China; ^3^Department of Center Laboratory, Wuhan Integrated TCM and Western Medicine Hospital, Wuhan, China; ^4^Hubei University of Traditional Chinese Medicine, Wuhan, China

## Abstract

**Objective:**

Sijunzi, Lizhong, and Fuzilizhong decoction were traditional Chinese classic formulations, which are widely used in clinical treatment, and the underlying mechanism is unclear. In this study, we aim to investigate the molecular mechanisms underlying the protective effects of Sijunzi, Lizhong, and Fuzilizhong on nonalcoholic fatty liver disease (NAFLD).

**Methods:**

Male Wistar rats were fed a high-fat diet for four weeks to induce NAFLD and were thereafter administered Sijunzi (8 g/kg/d), Lizhong (10 g/kg/d), or Fuzilizhong (10 g/kg/d) by gavage for four weeks. Hepatic damage, lipid accumulation, inflammation, autophagy, and peroxisome proliferator-activated receptor-*α* signaling were evaluated.

**Results:**

The high-fat diet-fed rats showed typical symptoms of NAFLD, including elevated levels of hepatic damage indicators, increased hepatic lipid deposition and fibrosis, severe liver inflammation, and prominent autophagy. Upon administration of Sijunzi, Lizhong, and Fuzilizhong, liver health was improved remarkably, along with ameliorated symptoms of NAFLD. In addition, NAFLD-suppressed peroxisome proliferator-activated receptor-*α* signaling was reactivated after treatment with the three types of decoctions.

**Conclusions:**

The results collectively signify the effective therapeutic and protective functions of Sijunzi, Lizhong, and Fuzilizhong against NAFLD and demonstrate the potential of Chinese herbal medication in mitigating the symptoms of liver diseases. *Novelty of the Work*. Traditional Chinese herbal medicine has been used for centuries to treat various diseases, but the molecular mechanisms of individual ingredients have rarely been studied. The novelty of our work lies in elucidating the specific signaling pathways involved in the control of NAFLD using three common Chinese herbal decoctions. We suggest that natural herbal formulations can be effective therapeutic agents to combat against NAFLD.

## 1. Introduction

The prevalence of nonalcoholic fatty liver disease (NAFLD) is increasing as overnutrition and obesity have become common among the worldwide population [1, 2]. NAFLD is characterized by necroinflammation and fat accumulation in the liver without significant alcohol consumption [3] and is closely associated with central abdominal obesity, diabetes, and dyslipidemia [4, 5]. The accumulation of triglycerides in hepatocytes could lead to the development of hepatic steatosis, whereas cellular stresses, such as oxidative stress, endotoxin stimulation, lipid peroxidation, and secretion of inflammatory cytokines can contribute to NAFLD [6]. Although the exact pathogenesis of NAFLD is not well understood, increased lipid accumulation, inflammation, autophagy, and oxidative stress are known to play important roles therein [7].

Traditional Chinese herbal medicine has been highlighted in the treatment of hepatic diseases such as NAFLD, as the use of natural herbal extracts is advantageous in preventing or mitigating undesired side effects. The theory of traditional Chinese medicine proposes that spleen dysfunction, damp-heat, and phlegm and stasis are the key manifestations of NAFLD. A special characteristic of Chinese herbal medicine is the preparation of a formulation containing several herbs to improve a set of abnormal syndromes associated with a disease. Herbal extracts contain a variety of natural compounds that target specific pathological pathways and provide therapeutic effects through a series of actions [8]. For example, the Sijunzi decoction (SJZ) has the functions of tonifying qi and strengthening the spleen, whereas the Lizhong (LZ) and Fuzilizhong (FZLZ) decoctions promote the replenishment of the spleen and warming of Yang. Previous studies have reported that SJZ, LZ, and FZLZ alleviated lipid peroxidation, liver fibrosis, and inflammatory reaction [9–11]. Of the major components of SJZ, LZ, and FZLZ decoctions, *Codonopsis pilosula* (Franch.) Nannf., *Atractylodes macrocephala* Koidz., and *Glycyrrhiza uralensis* Fisch. have demonstrated protective effects on the liver. Thus, it is essential to investigate the molecular mechanisms underlying the hepatoprotective effect of SJZ, LZ, and FZLZ against NAFLD.

A variety of factors regulate the synthesis, storage, and export of hepatic triglycerides and may thereby affect the magnitude of NAFLD. Among them, peroxisome proliferator-activated receptors (PPARs) are ligand-activated transcription factors belonging to the nuclear receptor superfamily, with three known subtypes: PPAR-*α*, −*γ*, and −*β*/*δ* [12]. PPAR-*α* is found mainly in the liver, kidney, and heart and controls the expression of genes involved in lipid and glucose metabolism [13]. The inhibition of PPAR-*α* was shown to increase cellular lipids and reduce the *β*-oxidation of fatty acids [14]. PPAR-*α* also negatively regulated acute phase response signaling pathways and proinflammatory reaction, as demonstrated in rodent models of NAFLD, atherosclerosis, systemic inflammation, and nonalcoholic steatohepatitis [15, 16]. Other factors contributing to NAFLD include sterol regulatory element-binding proteins (SREBPs), which are master transcription factors belonging to the basic helix-loop-helix leucine zipper family [17]. The activation of SREBP-1c triggers the expression of genes responsible for encoding enzymes that mediate hepatic glycogen and triglyceride synthesis, suggesting the potential role of SREBP-1c in the progression of NAFLD [18].

This study investigates the therapeutic effects of the three abovementioned decoctions used in Chinese herbal medicine (SJZ, LZ, and FZLZ) in a rat model of NAFLD. The extent of NAFLD and related indicators of disease progression were evaluated, and the possible involvement of the PPAR-*α* signaling pathway and SREBP-1c in the regulation of NAFLD progression was examined.

## 2. Materials and Methods

### 2.1. Drug Preparation

All herbal ingredients were purchased from Hubei Tianji Chinese Herbal Sliced Medicine Co., Ltd. SJZ is composed of dried roots of *Codonopsis pilosula* (Franch.) Nannf. (30 g, No: 201602010), and *Glycyrrhiza uralensis* Fisch. (12 g, No: 201602011). Dried rhizome of *Atractylodes macrocephala* Koidz. (18 g, No: 201602011) and *Poria cocos* (Schw.) Wolf (18 g, No: 201602004). Lizhong decoction (LZ) is composed of dried roots of *Codonopsis pilosula* (Franch.) Nannf. (30 g), and *Glycyrrhiza uralensis* Fisch. (12 g)., dried rhizome of *Atractylodes macrocephala* Koidz. (18 g), and dried rhizome of *Zingiber officinale* Rosc. (18 g, No: 20160127). FZLZ is composed of dried roots of *Codonopsis pilosula* (Franch.) Nannf. (30 g), *Glycyrrhiza uralensis* Fisch. (12 g), and *Aconitum carmichaelii* Debx. (Soak the *Aconitum carmichaelii* Debx. in water until it is completely immersed, then add water and boil for 4–6 hours. Remove the *Aconitum carmichaelii* Debx. and cut it open. The *Aconitum carmichaelii* Debx. should taste slightly tingly in the mouth. Then dry the slices completely) (18 g, No: 201601015). Dried rhizome of *Atractylodes macrocephala* Koidz. (18 g) and *Zingiber officinale* Rosc. (18 g).

For the preparation of SJZ and LZ, all ingredients were placed in a glass beaker and soaked in 400 mL of water for 30 min. The ingredients were then boiled at max heat, and the heat was reduced so that the decoction continued to simmer. After 20 min, the decoction was separated using a double-layer gauze sheet. The solid ingredients were then added to 350 mL of water, and the boiling steps were repeated. The filtered liquid portions from the two boiling steps were combined and concentrated accordingly. For the preparation of FZLZ, processed Chinese aconite was first soaked in 150 mL of water for 30 min, boiled at max heat, and simmered in low heat for 30 min. The other ingredients were placed in a glass beaker and soaked in 400 mL of water for 30 min. After boiling in max heat, the processed Chinese aconite was added, and the mixture was simmered at low heat for another 20 min. The decoction was separated using a double-layer gauze sheet. The solid ingredients were then added to 350 mL of water, and the boiling steps were repeated. The filtered liquid portions from the two boiling steps were combined and concentrated accordingly.

### 2.2. Detection of Active Ingredients in SJZ, LZ, and FZLZ

The active ingredients of SJZ, LZ, and FZLZ were detected using the ultrahigh performance liquid chromatography (UHPLC). A 100 *μ*l of aliquot concentrated SJZ, LZ, and FZLZ was extracted using 300 *μ*l of methanol via vortex mixing for 30 s. After ultrasound in an ice-water bath for 1 h, the specimen was centrifugated at 12000  rpm at room temperature for 10 min. Then a 5 *μ*l aliquot of the supernatant was harvested and analyzed by UHPLC (Agilent, CA, USA), equipped with a UPLC BEH C18 Column (1.7 *μ*m, 2.1 × 100 mm, Waters, Massachusetts, USA). The peak strength is usually proportional to the compound content. In the current work, the compounds in SJZ, LZ, and FZLZ with LQ.POS.B (stands for the signal strength of the drug component) >10000 were selected as active substances.

### 2.3. Animals and Treatments

All experimental procedures were approved by the Animal Ethics Committee of Wuhan Integrated TCM and Western Medicine Hospital (No. 42000600013948). Specific-pathogen-free male Wistar rats (8 weeks of age, 180–200 g) were obtained from the Animal Center of Hubei (Wuhan, China). The rats were maintained under conditions of constant temperature (21 ± 1°C) and humidity (50 ± 15%) in a 12 h/12h light/dark cycle, with free access to deionized water and fed irradiated disinfectant food. After one week of adaptive feeding, control rats (*n* = 8) were fed a standard chow diet and experimental rats (*n* = 32) were fed a high-fat diet for four weeks to induce NAFLD. The high-fat diet was composed of a mixture of two solutions, prepared as follows [19, 20]. Solution A contained a mixture of 25 g axungia dissolved by heating, 10 g of cholesterol, and 1 g of propylthiouracil tablets. After stirring, 25 mL of Tween 80 was added and mixed until use. Solution B contained 20 mL of propylene glycol in 30 mL of water. The solution was heated to 60°C, after which 2 g of sodium deoxycholate was added and mixed until use. Solutions A and B were mixed and 100 mL of water was added. The rats were given the same amount of standard chow diet and high-fat diet by gavage daily at 10 : 00 in the morning. Those fed the high-fat diet to induce NAFLD were randomly divided into four groups (*n* = 8 per group). Drug dosage was obtained based on clinical dose conversion according to the guide for dose conversion between animals and human [21] and treated as follows: no further treatment (NAFLD), SJZ (8 g/kg/d), LZ (8 g/kg/d), and FZLZ (10 g/kg/d). The medicine was administered by gavage for four weeks, and rats in the control and NAFLD groups were given the same volume of normal saline by gavage for four weeks.

### 2.4. Biochemical Analysis

At the end of treatment, the rats were anesthetized using 40 mg/kg pentobarbital after 12 h of fasting, and liver samples were collected. The levels of triglycerides (TG, product code: A110-1), total cholesterols (TC, A111-1), alanine aminotransferase (ALT, C009-2), high-density lipoprotein cholesterol (HDL-C, A112-2), and low-density lipoprotein cholesterol (LDL-C, A113-2) in the liver were determined using respective commercial kits (Nanjing Jiancheng Bioengineering Institute, Nanjing, China) following the manufacturers' instructions.

### 2.5. Oil Red O Staining

Fresh liver tissue was stored at −80°C for histopathological and molecular assays. Frozen sections were stained with Oil Red O (Bioswamp Life Science Lab, Wuhan, China) to investigate the architecture of the hepatic lipid droplets in the liver. Slides stained with Oil Red O were visualized with an Olympus microscope and images were captured with an Olympus digital camera.

### 2.6. Enzyme-Linked Immunosorbent Assay (ELISA)

Platelet-derived growth factor (PDGF, Biowamp, RA20454), fibroblast growth factor-2 (FGF-2, Biowamp, RA20438), vascular endothelial growth factor (VEGF, Biowamp, RA20124), interleukin (IL)-6 (RA20607, Biowamp), IL-12 (RA20651, Biowamp), and inducible NOS (iNOS, Biowamp, RA20644) were observed by ELISA. Standard solutions were prepared to generate a calibration curve of concentrations. Samples and enzymes were added to a test tube and incubated at 37°C according to the experimental instructions. The reaction was stopped 10 min after the color has appeared and the optical density was measured at 450 nm.

### 2.7. Immunohistochemistry, Masson's Trichrome, and Sirius Red Staining

Liver tissues were fixed in 10% formalin for 24 h, embedded in paraffin, and processed for immunohistochemistry, Masson's trichrome staining, and Sirius Red staining. For immunohistochemistry, the liver sections were incubated with rabbit anti-human *β*-catenin (ab32572, 1 : 500, Abcam, Cambridge, UK) and rabbit anti-mouse Axin 2 (ab32197, 1 : 1000, Abcam) at 4°C overnight. Thereafter, the samples were incubated with horseradish peroxidase-conjugated secondary antibodies, followed by staining with diaminobenzidine and counterstaining with hematoxylin.

### 2.8. Quantitative Reverse-Transcription Polymerase Chain Reaction (qRT-PCR)

Total RNA was extracted using Trizol (Invitrogen, Carlsbad, CA, USA), and cDNA was synthesized with the cDNA synthesis kit (639505, TaKaRa, Japan) according to the manufacturer's instructions. The amplification conditions were as follows: initial denaturation at 95°C for 3 min; 39 cycles of denaturation at 95°C for 5 s, annealing at 56°C for 10 s, and primer extension at 72°C for 25 s; and a final extension at 65°C for 5 s and 95°C for 50 s. The following primers were used: *β*-actin (internal control) forward, 5′-CGTTGACATCCGTAAAGAC-3′ and reverse, 5′-TAGGAGCCAGGGCAGTA-3'; transforming growth factor-*β*1 (TGF-*β*1) forward, 5′-AGGAGACGGAATACAGGG-3′ and reverse, 5′-GAGGAGCAGGAAGGGTC-3'; plasminogen activator inhibitor-1 (PAI-1) forward, 5′-CAGAGGTGGAAAGAGCC-3′ and reverse, 5′-GCCGTTGAAATAGAGGG-3'; collagen I forward, 5′-CTCAGCCCTCTGTGCCT-3′ and reverse, 5′-GAACCTTCGCTTCCATACT-3'; *α*-smooth muscle actin (*α*-SMA) forward, 5′-ACCATCGGGAATGAACG-3′ and reverse, 5′-TCAGCAATGCCTGGGTA-3'.

### 2.9. Western Blot

Proteins in liver tissue homogenates were extracted using ice-cold tissue lysis buﬀer, and the protein concentration was determined using a bicinchoninic acid protein assay (Bioswamp Life Science Lab). Proteins were loaded (30 *µ*g per lane) and separated by 10% sodium dodecyl sulfate-polyacrylamide gel electrophoresis. After electrophoresis, the proteins were transferred to polyvinylidene fluoride membranes. The membranes were then blocked in 5% nonfat milk for 1 h and incubated overnight at 4°C with rabbit primary antibodies against tumor necrosis factor-*α* (TNF-*α*, ab6671, 1 : 1000, Abcam), IL-1*β* (ab2105, 1 : 1000, Abcam), microtubule-associated protein light chain 3 (LC3B, ab51520, 1 : 3000, Abcam), PPAR-*α* (ab8934, 1 : 1000, Abcam), carnitine palmitoyltransferase-1 (CPT-1, ab83862, 1 : 1000, Abcam), CPT-2 (ab153869, 1 : 1000, Abcam), SREBP-1c (ab28481, 1 : 1000, Abcam), and *β*-actin (4970, Cell Signaling Technology, Danvers, USA). After three washes in phosphate-buffered saline/Tween 20, the membranes were incubated with horseradish peroxidase-conjugated goat anti-rabbit IgG secondary antibodies (PAB150011, 1 : 10000, Bioswamp Life Science Lab) for 1 h at 4°C and washed three times again with phosphate-buffered saline/Tween 20. The protein bands were visualized using an enhanced chemiluminescence system (GE Healthcare, Waukesha, WI, USA) and quantified using Quantity One software (Bio-Rad, Hercules, CA, USA).

### 2.10. Statistical Analysis

All data were analyzed with the statistical software SPSS 19.0 and expressed as the mean ± standard deviation (SD). Multigroup comparisons of the means were carried out by one-way analysis of variance with post hoc contrasts assessed by the Student-Newman-Keuls test. *P* < 0.05 was considered to be statistically significant.

## 3. Results

### 3.1. General Condition

We observed the general situation of the mice throughout the experiment, and all groups of mice did not restrict the supply of food and water. The results showed that compared with the control group, the weight of mice in the NAFLD group increased significantly (*P* < 0.05), compared with the NAFLD group, the weight of mice in the SJZ, LZ, and FZLZ groups decreased, and the weight of mice decreased gradually with the extension of the intervention time (*P* < 0.05). No rats died during the whole experimental intervention, suggesting that SJZ, LZ, and FZLZ have good safety (Table 1).

### 3.2. Identification of Active Ingredients in SJZ, LZ, and FZLZ

To study the effects of SJZ, LZ, and FZLZ on NAFLD, we performed LC-MS on SJZ, LZ, and FZLZ, respectively, and the results showed that there are 11 active substances in FZLZ (Ononin, Licoisoflavone A, Licoisoflavanone, Glyasperin C, Glabranin, Gancaonin B, Delcosine, Codonopsine, (S)-6-Gingerol, (S)-10-Gingerol, Liquiritigenin). SJZ have 8 active substances (Codonopsine, Ononin, Licoisoflavone A, Liquiritigenin, Syrigin, ergosterol, hederagenin, D-Camphene). There are 9 active substances in LZ (Ononin, Licoisoflavone A, Licoisoflavanone, Glyasperin C, Gancaonin B, Codonopsine, (S)-6-Gingerol, (S)-10-Gingerol, Liquiritigenin) (Table 2).

### 3.3. SJZ, LZ, and FZLZ Attenuated Hepatic Levels of NAFLD Indicators

**Figure 1:**
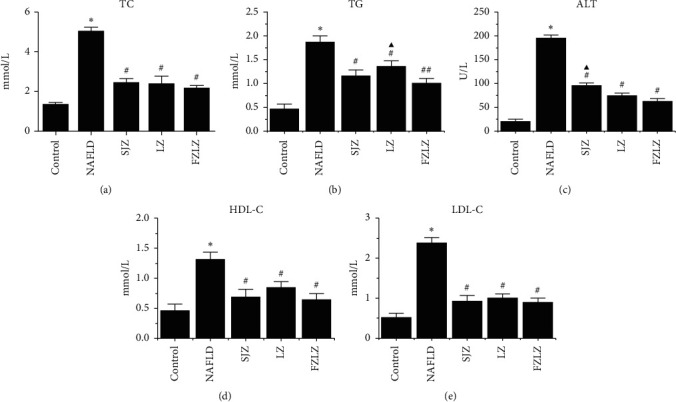
Effects of SJZ, LZ, and FZLZ on hepatic content of TC, TG, ALT, HDL-C, and LDL-C. ^*∗*^*P* < 0.05 vs. control; ^#^*P* < 0.05 vs. NAFLD; ^▲^*P* < 0.05 vs. FZLZ. SJZ: Sijunzi; LZ: Lizhong; FZLZ: Fuzilizhong; TC: total cholesterol; TG: triglycerides; HDL-C; high-density lipoprotein cholesterol; ALT: alanine aminotransferase; LDL-C: low-density lipoprotein cholesterol.

Rats subjected to a high-fat diet showed higher levels of TC, TG, ALT, HDL-C, and LDL-C (Figure.  1) in liver tissues compared to those fed a normal diet (*P* < 0.05), suggesting the successful induction of NAFLD-like symptoms model. Administration of SJZ, LZ, and FZLZ after NAFLD induction significantly reduced the hepatic levels of the abovementioned indicators to varying degrees (*P* < 0.05). In particular, FZLZ resulted in lower levels of ALT than did SJZ (*P* < 0.05) and led to further reduced TG content than did LZ (*P* < 0.05).

### 3.4. SJZ, LZ, and FZLZ Reduced Hepatic Lipid Accumulation

**Figure 2:**
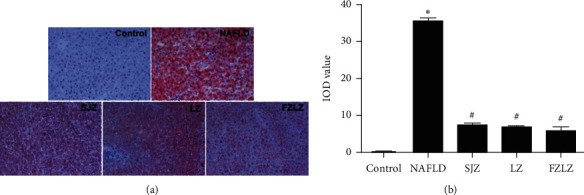
Histological changes of liver sections evaluated by Oil Red O staining (200× magnification). ^*∗*^*P* < 0.05 vs. control; ^#^*P* < 0.05 vs. NAFLD; ^▲^*P* < 0.05 vs. FZLZ. SJZ: Sijunzi; LZ: Lizhong; FZLZ: Fuzilizhong.

As is shown in Figure 2, the liver tissue of rats subjected to the high-fat diet (NAFLD) showed a tremendous amount of lipid accumulation. The administration of SJZ, LZ, and FZLZ accordingly reduced the degree of hepatic lipid deposition, as demonstrated by the lighter Oil Red O staining. FZLZ seemed to have a more prominent effect in reducing lipid accumulation than those of SJZ and LZ.

### 3.5. SJZ, LZ, and FZLZ Suppressed Liver Fibrosis

**Figure 3:**
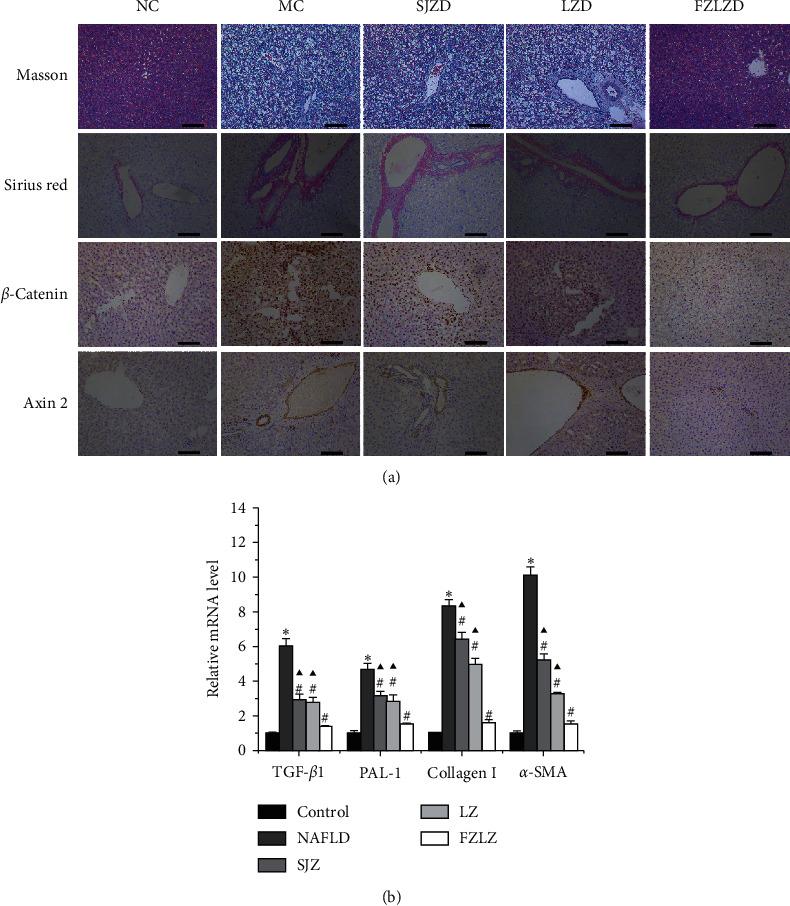
Effect of SJZ, LZ, and FZLZ on liver fibrosis. (a) Masson's trichrome staining, Sirius Red staining, and immunohistochemistry (*β*-catenin and Axin (2) were performed to detect liver fibrosis (scale bar = 50 *μ*m). (b) Relative mRNA levels of TGF-*β*1, PAI-1, collagen I, and *α*-SMA in the liver. ^*∗*^*P* < 0.05 vs. control; ^#^*P* < 0.05 vs. NAFLD; ^▲^*P* < 0.05 vs. FZLZ. SJZ: Sijunzi; LZ: Lizhong; FZLZ: Fuzilizhong; TGF-*β*1: transforming growth factor-*β*1; PAI-1: plasminogen activator inhibitor-1; *α*-SMA: *α α*-smooth muscle actin.

The degree of liver fibrosis was evaluated by Masson's trichrome and Sirius Red staining for collagen and connective tissues, respectively (Figure 3(a)). In the liver of rats fed a high-fat diet (NAFLD), Masson's trichrome staining revealed significant areas of blue staining indicative of distinct collagen deposition and fibrosis, while Sirius Red staining showed a similar phenomenon for connective tissues. When NAFLD-induced rats were treated with SJZ, LZ, and FZLZ, their livers showed remarkable reductions in fibrotic areas. Immunohistochemistry further demonstrated that the hepatic expression of *β*-catenin and Axin 2, which was significantly elevated by the high-fat diet, was suppressed by SJZ, LZ, and FZLZ (Figure 3(a)). In addition, NAFLD induced a significant increase in the mRNA expression of TGF-*β*1, PAI-1, collagen I, and *α*-SMA (Figure 3(b)) and promoted the secretion of PDGF, FGF-2, and VEGF (Table 3), whereas these indicators were all downregulated by SJZ, LZ, and FZLZ (*P* < 0.05), with FZLZ exhibiting the greatest effect in suppressing liver fibrosis (*P* < 0.05).

### 3.6. SJZ, LZ, and FZLZ Mitigated NAFLD-Associated Inflammation and Autophagy

**Figure 4:**
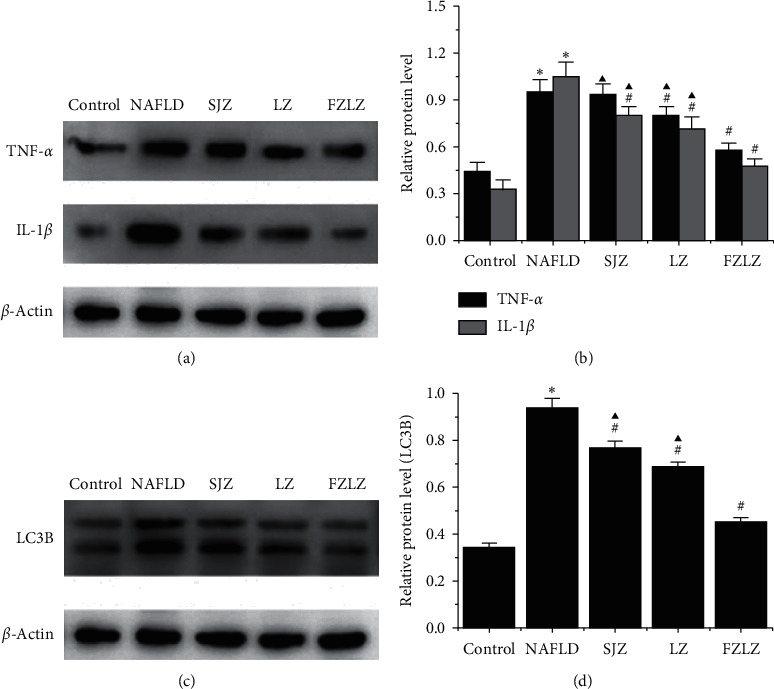
Effect of SJZ, LZ, and FZLZ on proteins associated with inflammation and autophagy. (a) Western blot and (b) quantification of relative expression of TNF-*α* and IL-1*β* in the liver. (c) Western blot and (d) quantification of relative expression of LC3B in the liver. ^*∗*^*P* < 0.05 vs. control; ^#^*P* < 0.05 vs. NAFLD; ^▲^*P* < 0.05 vs. FZLZ. SJZ: Sijunzi; LZ: Lizhong; FZLZ: Fuzilizhong; TNF-*α*: tumor necrosis factor-*α*; IL-1*β*: interleukin-1*β*; LC3B: microtubule-associated protein light chain 3.

To determine the effect of SJZ, LZ, and FZLZ on liver autophagy, the expression of several proteins associated with inflammation (IL-6, IL-12, iNOS, TNF-*α*, and IL-1*β*) and autophagy (LC3B) was detected in liver tissues, as summarized in Table 2 and Figure 4. The expression levels of IL-6, IL-12, iNOS (Table 4), TNF-*α*, IL-1*β* (Figures 4(a) and 4(b)), and LC3B (Figures 4(c) and 4(d)) in the liver of NAFLD-induced rats were higher than those in control rats (*P* < 0.05). These proteins were downregulated by the administration of SJZ, LZ, and FZLZ (*P* < 0.05) (except for SJZ, which did not seem to affect TNF-*α* expression), with FZLZ exerting a greater effect than those of SJZ and LZ (*P* < 0.05). The results revealed that the three decoctions had suppressive effects on liver inflammation and autophagy induced by NAFLD.

### 3.7. Effect of SJZ, LZ, and FZLZ on PPAR Pathway

**Figure 5:**
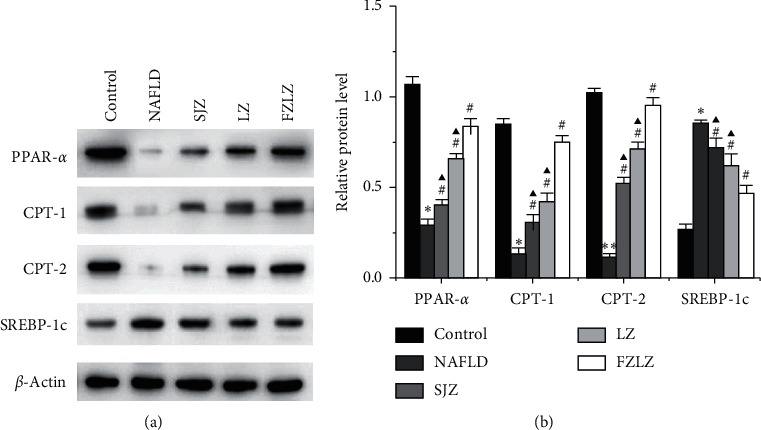
Effect of SJZ, LZ, and FZLZ on the PPAR pathway. (a) Western blot and (b) quantification of relative protein expression of PPAR-*α*, CPT-1, CPT-2, and SREBP-1c in the liver. ^*∗*^*P* < 0.05 vs. control; ^#^*P* < 0.05 vs. NAFLD; ^▲^*P* < 0.05 vs. FZLZ. SJZ: Sijunzi; LZ: Lizhong; FZLZ: Fuzilizhong; PPAR-*α*: peroxisome proliferator-activated receptor-*α*; CPT-1: carnitine palmitoyltransferase 1; CPT-2: carnitine palmitoyltransferase 2; SREBP-1c: sterol regulatory element-binding protein-1c.

The regulatory role of the PPAR pathway in NAFLD was examined by western blot detection (Figure 5(a)) and quantification (Figure 5(b)) of proteins relevant to the PPAR signaling pathway. In rats fed a high-fat diet (NAFLD), the protein levels of PPAR-*α*, CPT-1, and CPT-2 were downregulated while that of SREBP-1c was elevated (*P* < 0.05), suggesting the inhibition of PPAR-*α* and activation of SREBP-1c. Upon administration of SJZ, LZ, and FZLZ, the expression of PPAR-*α*, CPT-1, and CPT-2 was upregulated while that of SREBP-1c was reduced (*P* < 0.05) to various extents, indicating that the three decoctions ameliorated the symptoms of NAFLD by promoting PPAR-*α* and inhibiting SREBP-1c.

## 4. Discussion

The aim of the present study was to reveal the molecular mechanisms underlying the protective effects of SJZ, LZ, and FZLZ against NAFLD. NAFLD is identified by the overaccumulation of lipids, triglycerides, and cholesterol [22] and is associated with lipid metabolic disorders, inflammatory cytokine secretion, oxidative stress, autophagy, and changes in related signaling pathways [23]. Another pathological feature of NAFLD is hepatic fibrosis, which is considered to signify the end stage of chronic liver diseases and often leads to cirrhosis [24]. A variety of factors, including PDGF, FGF-2, VEGF, *β*-catenin, Axin 2, TGF-*β*1, PAI-1, collagen I, and *α*-SMA, are key mediators of hepatic fibrosis [25, 26]. As expected, rats fed a high-fat diet for four weeks displayed a series of NAFLD-associated symptoms, including high hepatic TG, TC, ALT, HDL-C, and LDL-C levels (Figure 1), lipid accumulation (Figure 2), and fibrosis (Figure 3) in liver tissues. The administration of SJZ, LZ, and FZLZ accordingly attenuated the levels of liver damage induced by NAFLD.

Recently, increasing evidence has shown the vital role of autophagy in the development and progression of NAFLD [27]. Autophagy, a cell survival mechanism, is essential for the regulation of hepatic lipid metabolism. Autophagic dysfunction may increase lipid accumulation in the liver, thus participating in the emergence and progression of NAFLD [28]. The upregulation of the autophagy-lysosomal pathway might promote lysosomal lipid absorption and eventually alleviate hyperlipidemia-associated symptoms [29]. Liu et al. reported that the hepatic levels of TNF-*α*, IL-1*β*, and LC3B in mice fed a high-fat diet were upregulated as a result of hyperlipidemia. However, liver inflammation was alleviated by rutin as evidenced by the downregulation of TNF-*α* and IL-1*β* in liver tissues, and autophagy was reduced as demonstrated by the downregulation of LC3B in liver tissues [30]. These observations are in agreement with the results of the current study, whereby SJZ, LZ, and FZLZ suppressed liver inflammation due to NAFLD, as suggested by the decrease in IL-6, IL-12, iNOS, TNF-*α*, and IL-1*β* expression in liver tissues (Table 2 and Figure 4). Furthermore, the expression of LC3B in liver tissues, which reflects hepatic autophagy, was upregulated by NAFLD but downregulated by SJZ, LZ, and FZLZ (Figure 4).

Lipid homeostasis is controlled by the delicate balance between lipid synthesis and metabolism. Increased uptake of fatty acids in the liver could lead to lipid accumulation, and lipid-related diseases often involve the dysregulation of lipid homeostasis. PPAR-*α*, which is associated with hepatic lipid accumulation, is strongly expressed in the heart, intestinal mucosa, kidney, brown fat, skeletal muscle, and liver [31]. It regulates the expression of genes involved in lipoprotein metabolism, fatty acid oxidation, cholesterol catabolism, ketogenesis, and gluconeogenesis [32]. The decrease in PPAR-*α* often results in hyperlipidemia, whereas its upregulation leads to increased *β*-oxidation activity of fatty acids and lower hepatic TG levels [33, 34]. The present study showed that the downregulation of PPAR-*α* due to NAFLD was counteracted by SJZ, LZ, and FZLZ. In addition, NAFLD caused a decrease in the protein levels of CPT-1 and CPT-2 and an increase in that of SREBP-1c, whereas the three decoctions counteracted the effects of NAFLD. CPT-1 and CPT-2, two mitochondrial membrane-associated enzymes, together with control fatty acid influx into mitochondria where *β*-oxidation occurs [35]. Meanwhile, SREBP-1c plays a pivotal role in the development and pathogenesis of NAFLD [36], and the downregulation of SREBP-1c reportedly suppressed de novo fatty acid synthesis in fatty acid overload [37]. Herein, the reduction of hepatic lipid accumulation mediated by SJZ, LZ, and FZLZ was accompanied by the activation of PPAR-*α* signaling and the inhibition of SREBP-1c.

In this study, we found that SJZ, LZ, and FZLZ can effectively alleviate hepatic lipid accumulation and fibrosis and thus play a role in the treatment of NAFLD. According to the TCM theory, FZLZ is better than LZ in treating fatty liver with Yang deficiency (aversion to cold, pale complexion, etc.), while SJZ is better than LZ in treating fatty liver with qi deficiency (limb weakness, dizziness, pale complexion, etc.). The common active substances of SJZ, LZ, and FZLZ were Ononin, Licoisoflavone A, Codonopsine, and Liquiritigenin by HPLC analysis, suggesting that Ononin, Licoisoflavone A, Codonopsine, and Liquiritigenin may be effective active substances for the treatment of NAFLD. In this study, we mainly studied the effects of FZLZ, SJZ, and LZ on NAFLD and its potential mechanisms, and we will further study the effects of Ononin, Licoisoflavone A, Codonopsine, and Liquiritigenin on NAFLD in the next experiments.

Taken together, the results summarized here indicate that SJZ, LZ, and FZLZ exert protective effects against liver damage by attenuating hepatic lipid accumulation and fibrosis. The three decoctions also ameliorated the symptoms of NAFLD by mitigating liver autophagy and regulating PPAR-*α* and its downstream target enzymes.

## Figures and Tables

**Table 1:** Body weight (g).

	7 d	14 d	21 d	28 d
Control	200 ± 9	203 ± 6	208 ± 2	207 ± 7
NAFLD	280 ± 6*∗*	281 ± 6*∗*	289 ± 6*∗*	285 ± 8*∗*
SJZ	262 ± 8^#,▲^	243 ± 4^#,▲^	238 ± 4^#,▲^	230 ± 4^#^
LZ	258 ± 8^#,▲^	244 ± 6^#,▲^	234 ± 4^#,▲^	228 ± 3^#^
FZLZ	260 ± 10^#^	238 ± 4^#^	226 ± 3^#^	225 ± 5

*Note*.*∗P* < 0.05 vs. control; #*P* < 0.05 vs. NAFLD; ▲ *P* < 0.05 vs. FZLZ.

**Table 2:** Active ingredients in SJZ, LZ, and FZLZ.

	Description	*m*/*z*.	Retention time (min)	LQ.POS.B
FZLZ	Codonopsine	268	2	244231
	(S)-6-Gingerol	317	10	176871
	Delcosine	454	4	173698
	Ononin	431	7	32579
	(S)-8-Gingerol	345	12	23434
	Glyasperin C	357	11	22229
	Licoisoflavanone	355	11	14754
	Licuroside	551	7	14386
	Aconifine	662	9	14237
	Gancaonin B	369	10	11913
	(S)-10-Gingerol	373	13	11716
SJZ	Codonopsine	268	2	277264
	Ononin	431	7	58366
	Licoisoflavone A	353	11	82933
	Liquiritigenin	255	7	26996
	Syrigin	983	8	76912
	ergosterol	367	10	65465
	hederagenin	417	7	57386
	D-Camphene	367	10	18561
LZ	(S)-6-Gingerol	317	10	248214
	(S)-10-Gingerol	373	13	17508
	Codonopsine	268	2	258584
	Ononin	431	7	53659
	Glyasperin C	357	11	23025
	Licoisoflavanone	355	11	20824
	Gancaonin B	369	10	11995
	Licoisoflavone A	353	11	77599
	Liquiritigenin	255	7	19091

**Table 3:** Effect of SJZ, LZ, and FZLZ on PDGF, FGF-2, and VEGF content in the liver.

	PDGF (ng/mL)	FGF-2 (pg/mL)	VEGF (pg/mL)
Control	1.38 ± 0.01	72.4 ± 0.61	208 ± 3
NAFLD	4.14 ± 0.01*∗*	250 ± 0.67*∗*	622 ± 3*∗*
SJZ	2.54 ± 0.02^#,▲^	163 ± 1^#,▲^	418 ± 3^#,▲^
LZ	2.60 ± 0.01^#,▲^	154 ± 1^#,▲^	434 ± 4^#,▲^
FZLZ	2.16 ± 0.01^#^	138 ± 1^#^	360 ± 2^#^

*Note.* ^*∗*^ *P* < 0.05 vs. control; ^#^*P* < 0.05 vs. NAFLD; ^▲^*P* < 0.05 vs. FZLZ.

**Table 4:** Effect of SJZ, LZ, and FZLZ on IL-6, IL-12, and iNOS content in the liver.

	IL-6 (pg/mL)	IL-12 (pg/mL)	iNOS (ng/mL)
Control	64.7 ± 1.2	96.4 ± 1	0.51 ± 5.23
NAFLD	161 ± 1*∗*	206 ± 1*∗*	1.41 ± 4.75*∗*
SJZ	116 ± 1^#,▲^	141 ± 1^#,▲^	0.78 ± 2.81^#,▲^
LZ	109 ± 0^#,▲^	146 ± 1^#,▲^	0.82 ± 2.08^#,▲^
FZLZ	98.6 ± 0.5^#^	129 ± 0^#^	0.71 ± 1.54^#^

*Note.* ^*∗*^ *P* < 0.05 vs. control; ^#^*P* < 0.05 vs. NAFLD; ^▲^*P* < 0.05 vs. FZLZ.

## Data Availability

The datasets used and/or analyzed during the current study are available from the corresponding author on reasonable request.
